# A quantitative assessment of termination of sexual violence-related pregnancies in eastern Democratic Republic of Congo

**DOI:** 10.1186/s13031-016-0073-x

**Published:** 2016-04-06

**Authors:** Shada A. Rouhani, Jennifer Scott, Gillian Burkhardt, Monica A. Onyango, Sadia Haider, Ashley Greiner, Katherine Albutt, Michael VanRooyen, Susan A. Bartels

**Affiliations:** Harvard Humanitarian Initiative, Cambridge, MA USA; Department of Emergency Medicine, Brigham and Women’s Hospital, Boston, MA USA; Harvard Medical School, Boston, MA USA; Department of Obstetrics and Gynecology, Beth Israel Deaconess Medical Center, Boston, MA USA; Division of Women’s Health, Brigham and Women’s Hospital, Boston, MA USA; Department of Obstetrics and Gynecology, Boston University School of Medicine, Boston, MA USA; Department of Global Health, Boston University School of Public Health, Boston, MA USA; Department of Obstetrics and Gynecology, University of Illinois at Chicago, Chicago, USA; Department of Emergency Medicine, Beth Israel Deaconess Medical Center, Boston, MA USA; Department of Surgery, Massachusetts General Hospital, Boston, MA USA; Harvard School of Public Health, Boston, MA USA; Department of Emergency Medicine, Queen’s University, Kingston, Canada

**Keywords:** Gender based violence, Sexual violence, Termination, Pregnancy, Democratic Republic of Congo

## Abstract

**Background:**

Sexual violence is prevalent in eastern Democratic Republic of Congo (DRC), and has resulted in sexual violence-related pregnancies (SVRPs). Despite restrictive laws, women may seek to terminate SVRPs; however, there are limited data on termination of SVRPs.

**Methods:**

A mixed methods study was conducted in 2012 in Bukavu, DRC. Adult women who self-reported an SVRP and termination of that SVRP were recruited using respondent-driven sampling (RDS). Trained female interviewers verbally administered a quantitative survey to all participants and a semi-structured qualitative survey to a subset. Quantitative data on characteristics and complications of pregnancy termination, including mental health outcomes, were analyzed using SAS.

**Results:**

In total, 86 women completed quantitative surveys. Most SVRPs (93 %) involved two or more assailants; 73 % occurred while in captivity. Most women (82 %) terminated the SVRPs at 3 months gestation or earlier; 79 % reported one attempt at pregnancy termination and 21 % more than one attempt. The most common methods of termination were an oral medicine (55 %) or herb (35 %); cimpokolo (31 %) and quinine (18 %) were most frequently reported. These methods were accessed through friends (37 %), healthcare providers (18 %), family (16 %), or self-obtained (12 %). Following the termination, 79 % of women reported subsequent physical symptoms, including abdominal pain (74 %), bleeding (47 %), vaginal discharge (35 %) and fever (18 %); 44 % sought medical care for their symptoms. Varied emotional responses to the termination were reported and included relief (34 %), anxiety (21 %), anger (19 %), guilt (19 %), and regret (10 %). At the time of the study, many women met symptom criteria for post-traumatic stress disorder (57 %), depression (50 %), and generalized anxiety disorder (33 %).

**Conclusion:**

Most women terminated SVRPs using medications or herbs not recognized as evidence-based methods of pregnancy termination and sought these methods outside of the formal healthcare sector. These data suggest that access to safe abortion methods is needed for women with SVRPs in DRC. Physical symptoms and emotional reactions related to the termination varied. While it is not possible to differentiate the impacts of sexual violence, SVRP, and pregnancy termination on mental health outcomes, the findings highlight the complex needs of women with SVRPs and opportunities for integrative health services.

## Background

Sexual violence has been prevalent throughout two decades of conflict in eastern Democratic Republic of Congo (DRC), with an estimated 40 % of the female population reporting lifetime experiences of sexual assault [1]. Unintended pregnancies are one of the many public health, politico-legal, and socioeconomic consequences of sexual violence [2]. Although protocols for post-assault medical care exist [3, 4], including the prevention and management of sexual violence-related pregnancies (SVRPs) [3, 5, 6], many women cannot or do not seek care after sexual violence [7–9]. Even if post-assault care is accessed, emergency contraception may not be available [10] or effective, given delays in seeking care [7, 11]. In eastern DRC, up to 17 % of sexual violence survivors report SVRPs [1, 12, 13]; However, there are limited data about how many SVRPs are terminated, what methods of termination are used, or about the medical, psychosocial, or legal outcomes of pregnancy terminations in this context.

Prior research suggests that nearly half of women with SVRPs seek or undergo termination of the pregnancy [9, 14, 15]. However, in DRC, as in other conflict and post-conflict settings, access to comprehensive and safe abortion and post-abortion care is limited [16–19]. Pregnancy termination is highly restricted in DRC, even within the context of sexual violence [2, 20]. Technically, the DRC has signed and ratified the *Maputu Protocol,* which permits pregnancy terminations in cases of rape or incest. However, the 1982 DRC Penal Code stipulates that abortions are illegal and subject to 5 to 15 years imprisonment [18]. Limited access to safe abortion care increases maternal morbidity and mortality [17, 21] and limits the reporting of termination outcomes [22, 23]. The often clandestine nature of terminations in this context poses challenges to understanding the number of women who terminate SVRPs, the methods of termination, or the outcomes of the terminations. Further evidence is needed in order to provide comprehensive care and support to sexual violence survivors.

To address evidence gaps around the termination of SVRPs in eastern DRC, this study aimed to: 1) understand what methods are used to terminate SVRPs in this context, 2) describe complications and consequences related to termination of SVRPs, and 3) assess mental health outcomes among women who terminated SVRPs.

## Methods

This mixed methods study was conducted in Bukavu, South Kivu Province, DRC, in October-November 2012.

### Respondent driven sampling

Participants were recruited using respondent-driven sampling (RDS). RDS uses controlled peer-to-peer recruitment to sample hard-to-reach populations [24, 25]. Initial seed participants are purposively chosen and subsequently recruit eligible peers to participate, and these peers then recruit additional participants. Recruitment patterns are carefully tracked using uniquely numbered coupons; analysis of these patterns and social network size helps to identify and correct for recruitment biases in the final data analysis. When sufficient recruitment is reached, the data from an RDS sample can approximate an unbiased estimate of population parameters [24–26].

### Participant recruitment and study procedures

Detailed survey methodology for this study has been previously published [27]. Briefly, two study groups were recruited: 1) women raising children from SVRPs (parenting group), and 2) women who terminated SVRPs (termination group). A woman aged 18 or older was eligible for participation if she self-identified as a sexual violence survivor since the start of the conflict (~1996), conceived an SVRP, and terminated the SVRP (termination group) or was currently raising a child from an SVRP (parenting group). Quantitative data from the parenting group were previously published [28, 29]. Quantitative data from the termination group are presented here.

In collaboration with local partners, 18 women who met study criteria were selected and interviewed as initial participants (eight in the parenting group, eight in the termination group and two who met study criteria for both the parenting and termination groups). Each initial participant could recruit up to three other eligible women with SVRPs, who could each recruit three others, and so on. Cross recruitment between the parenting and termination groups was permitted, although each group had dedicated surveys and data analysis plans. On arrival to the study office with a valid coupon, each recruited participant answered standardized screening questions to confirm study eligibility. After obtaining verbal informed consent, trained local female research assistants verbally administered the quantitative survey in a private setting. A subsample of participants was invited to complete a semi-structured interview for the qualitative portion of the study; the qualitative data from the termination group are presented separately (submitted to Conflict and Health). Headscarves (approximately $1 USD each) were provided as compensation for the participants’ time; transportation costs (up to $8 USD) were reimbursed directly to the taxi drivers.

### Ethics, consent and permissions

The Harvard School of Public Health provided institutional review board approval, the Provincial Minister of Health and Medical Inspector provided local approval, and a community advisory board provided study oversight. Verbal informed consent was obtained from all participants. Two trained psychosocial assistants were available for on-site counseling and participants received a referral card for medical and/or mental health care. All interviewers completed a 6-day training and had prior research experience and/or experience working with sexual violence survivors. Privacy was strictly protected; identifying information was not collected and no study-related documents disclosed the sensitive nature of the study. Electronic devices and data were encrypted and password protected.

### Instruments

The quantitative survey was designed with local collaborators and then refined through pilot testing and cognitive interviewing. It was written in English, translated into Swahili, and back translated by a second person. A panel resolved translation discrepancies. The survey was verbally administered in Swahili and responses were recorded on electronic handheld devices using KOBO® technology [30]. If an electronic device was not available, paper surveys were used and the data was retrospectively entered.

Demographic characteristics including age, place of origin, current residency, marital status, ethnicity, and religion, were self-reported by participants. The survey assessed sexual assault characteristics and details about how the pregnancy was terminated, who provided assistance with the termination, as well as physical symptoms, psychological symptoms, and socioeconomic outcomes following the termination.

Mental health outcomes were measured with instruments previously used in conflict settings and among survivors of violence [1, 31–33]. Using a cut-off score of ≥3, validated short forms of the Patient Health Questionnaire PHQ-9 (PHQ-2) [34, 35] and the Generalized Anxiety Disorder GAD-7 (GAD-2) [36] assessed symptoms of depression and anxiety, respectively, during the two-weeks preceding survey administration. Using a cut-off score of ≥50, the Post Traumatic Stress Disorder (PTSD) Checklist-Civilian Version (PCL-C) assessed symptoms of PTSD during the 4 weeks preceding survey administration [37]. Women were also asked about suicidal ideation and suicide attempts since the start of the war (~1996).

### Definitions

Sexual violence was defined as “intercourse against your will, being forced to undress, molestation, and other unwanted sexual acts”. An SVRP was any pregnancy self-reported to have been conceived as a result of sexual violence. A perpetrator was any person who inflicted sexual violence. Pregnancy termination was any self-reported attempt that interrupted an SVRP.

### Statistical analysis

If an RDS study achieves sufficient recruitment, the data may approximate an unbiased estimate of population parameters [24–26]. The desired sample size for the termination group was 636, assuming a design effect of two [38] and 50 % prevalence of depression [1]. However, due to security concerns, the study stopped prematurely after 4 weeks of data collection; as a result, the termination group did not reach sufficient recruitment. Therefore, these data resemble a snowball sample and no attempt was made to correct for recruitment biases, as is typically done in an RDS analyses. Data were analyzed in SAS, version 9.3. Mental health outcomes were compared with chi-squared tests.

## Results

In total, 86 women completed the quantitative survey. The demographic characteristics of participants are provided in Table 1. The majority represented a single ethnic group, Bashi (87 %). Participants self-identified as Roman Catholic (52 %) or Protestant (48 %) and the majority (52 %) did not have any formal education. Regarding the sexual assault that led to the SVRP, 93 % of participants reported two or more perpetrators, and 73 % reported the pregnancy occurred while in captivity (Table 2). The majority of perpetrators were reported to be from armed groups (95 %).

**Table 1:** Demographic characteristics of women who terminated sexual violence-related pregnancies in Eastern Democratic Republic of Congo

Ethnic group	
Bashi	75 (87 %)
Belega	7 (8 %)
Babembe	1 (1 %)
Bahavu	1 (1 %)
Bavira	1 (1 %)
Marital status	
Married or living with partner	31 (36 %)
Widowed or husband missing	27 (31 %)
Divorced or separated	18 (21 %)
Never married	10 (12 %)
Religion	
Roman Catholic	45 (52 %)
Protestant	41 (48 %)
Occupation	
Unemployed	30 (35 %)
Farmer	25 (29 %)
Market vendor	14 (16 %)
Housewife/homemaker	12 (14 %)
Other	5 (6 %)
Education	
None	44 (52 %)
Any primary school	27 (32 %)
Any secondary school	13 (15 %)
Meals per day	
1	70 (81 %)
2	16 (19 %)
3	0 (0 %)

**Table 2:** Lifetime experiences of sexual violence and characteristics of the sexual assault leading to an SVRP

Lifetime experiences of sexual violence	
One episode	67 (78 %)
Two episodes	16 (19 %)
Three episodes	3 (3 %)
Number of assailants	
One	6 (7 %)
Two or more	80 (93 %)
Pregnancy occurred while in captivity	
Yes	62 (73 %)
No	23 (27 %)
Type of perpetrator	
Military or armed group	81 (94 %)
Civilian	4 (5 %)
Both military or armed assailants and civilian assailants	1 (1 %)

Characteristics of the pregnancy terminations are provided in Table 3. The majority of participants terminated in the second or third month of pregnancy; however 17 % of women reported terminating between the fourth and sixth month of pregnancy. In assessing knowledge among participants of how and where to terminate a pregnancy, 19 % of participants reported already having this knowledge at the time the SVRP was recognized. Others reported receiving advice from friends (35 %), family members (14 %), and village chiefs or elders (10 %). The majority (79 %) terminated the SVRP after one attempt, while a minority required two (16 %) or three (5 %) attempts to terminate the pregnancy.

**Table 3:** Characteristics of the SVRP terminations

Gestational age in months at time of termination	
1	3 (3 %)
2	17 (20 %)
3	51 (59 %)
4	12 (14 %)
5	2 (2 %)
6	1 (1 %)
Who told the participant where to go for the termination	
Friend	30 (35 %)
No one told me	16 (19 %)
Family member	12 (14 %)
Village chief/elder	9 (10 %)
Community member	7 (8 %)
Doctor/nurse/midwife	6 (7 %)
Traditional healer	5 (6 %)
Other	1 (1 %)
Number of termination attempts	
One	68 (79 %)
Two	14 (16 %)
Three	4 (5 %)
Method used to terminate the SVRP^a^	
Took a medicine by mouth	47 (55 %)
Took a herb by mouth	39 (35 %)
Placed medicine in vagina	6 (7 %)
Placed herb in vagina	1 (1 %)
Placed medicine in rectum	4 (5 %)
Placed herb in rectum	1 (1 %)
Injected a medicine	2 (2 %)
Used an instrument or procedure	1 (1 %)
Participated in ritual/ceremony	0 (0 %)
Other	1 (1 %)
Specific herb or medication used^ab^	
Cimpokolo	26 (31 %)
Quinine	15 (18 %)
Multiple methods (including multiple herbs)	8 (10 %)
Sombe/Cassava	5 (6 %)
Mutuzo	2 (2 %)
Mayanibuchungu	2 (2 %)
Root of Sorghum	2 (2 %)
Aloe Vera	2 (2 %)
Misoprostol (Cytotec)	1 (1 %)
Curretage	1 (1 %)
Washing with dirty water	1 (1 %)
Luungusu	1 (1 %)
Cimbehe	1 (1 %)
Cilula/Cigaka	1 (1 %)
Salt	1 (1 %)
Papaya	1 (1 %)
Who did the procedure or gave the medication or herb?^c^	
Friend	32 (37 %)
Family member	14 (16 %)
Community elder/chief	13 (15 %)
Participant herself	12 (14 %)
Traditional healer	10 (12 %)
Physician or nurse	10 (12 %)
Community member	9 (10 %)
Spouse	5 (6 %)
Midwife	5 (6 %)
Pharmacist	4 (5 %)
Perpetrator	3 (3 %)
Don’t remember or don’t know	3 (3 %)
What was paid for the termination	
Nothing	61 (71 %)
Money	17 (20 %)
Other (fabric (2), hens (1), worked in field (3))	6 (7 %)
Don’t know	2 (2 %)
Of those who paid money for the termination (*n* = 17), how much money was paid (USD)?	
$1–$10	13 (77 %)
$11–$25	2 (12 %)
$25–$50	1 (6 %)
$51–100	1 (6 %)
Of those who paid money for the termination (*n* = 17), what was the source of the money?	
Had own money	5 (29 %)
Friend or family	5 (29 %)
Sold items	3 (18 %)
Other	1 (6 %)
No response or don’t remember	3 (18 %)

^a^participants could report more than 1 method, so percentages may add to more than 100 %

^b^This question was free response

^c^participants could report more than 1 response, so percentages may add to more than 100 %

Most women terminated the SVRP by taking a medication (55 %) or an herb (35 %) orally. A minority reported vaginal (7 %) or rectal (5 %) administration of a medication or vaginal (1 %) or rectal (1 %) administration of an herb. The most commonly reported medication was quinine and the most commonly reported herb was cimpokolo, known by the scientific name Phytolacca dodecandra L’Hérit [39, 40]. Only one participant reported use of a procedure or instrument as a method of pregnancy termination. A minority (10 %), reported using more than one method of termination, including more than one herb or medication. Only 12 % of participants reported seeing a physician or nurse for the medication or procedure, with an additional 6 % seeing a midwife. Most women (71 %) did not pay for the termination, while 20 % paid money and 7 % reported non-monetary means of payment (hens, fabric, work in fields).

The reported symptoms associated with pregnancy terminations are listed in Table 4. Most women (79 %) reported physical symptoms or complications. Of those reporting physical symptoms, the most common were abdominal pain (74 %), bleeding (47 %), vaginal discharge (35 %), and fever (18 %). The severity of these symptoms was not assessed, but nearly half of those with physical symptoms reported seeking medical care for the symptoms. The most common emotional response was relief, reported by 34 % of participants, followed by anxiety (21 %), anger (19 %), and guilt (19 %); participants could report more than one response. In this sample, 80 % of participants reported that legal consequences could arise as a result of terminating an SVRP. In addition, 6 % reported that they had appeared in court after terminating an SVRP. Further information about the role of the courts or about specific legal consequences was not collected.

**Table 4:** Health-related consequences of terminating the SVRP

Had physical symptoms or complications as a result of the termination	
Yes	68 (79 %)
No	18 (21 %)
Type of physical symptom or complication^a^	
Abdominal pain	50 (74 %)
Bleeding	32 (47 %)
Vaginal discharge	24 (35 %)
Fever	12 (18 %)
Needed hospitalization	3 (4 %)
Continuation of pregnancy	1 (1 %)
Infertility	1 (1 %)
Of those reporting physical complications (*n* = 68), was care sought for those complications?	
Yes	30 (44 %)
No	38 (56 %)
Where was care sought^a^	
Dispensary/health center	14 (47 %)
Hospital	6 (20 %)
Doctor/health worker	5 (17 %)
Pharmacy	2 (7 %)
Community member	1 (3 %)
Traditional healer	1 (3 %)
Same person who did termination	1 (3 %)
Emotional and psychosocial response of participant to the termination of the SVRP^a^	
Relief	29 (34 %)
Anxiety	18 (21 %)
Anger	16 (19 %)
Guilt	16 (19 %)
None	14 (16 %)
Regret	9 (10 %)
Shame	5 (6 %)
Fear about legal consequences	4 (5 %)
Fear about others finding out	3 (4 %)
Change in sleep	3 (3 %)
Sadness	2 (2 %)
Other^b^	3 (3 %)

^a^participants could report more than 1 response, so percentages may add to more than 100 %

^b^Other responses, each reported by one participant: change in eating, stigma from friends/family/community, forced to leave home/community, caused trouble for other members of family, other

Analysis of mental health outcomes showed that at the time of survey administration, many respondents met symptom criteria for PTSD (57 %), depression (50 %), and generalized anxiety disorder (33 %). Over one quarter reported current or past suicidal thoughts or attempts since the start of the war (~1996) (Table 5). In chi squared testing, mental health outcomes did not vary significantly among those who reported physical symptoms or varying psychosocial responses following the termination (all *p*-values > 0.05).

**Table 5:** Mental health outcomes at the time of the survey among women who terminated SVRPs

Met symptom criteria for depression	
Yes	43 (50 %)
No	43 (50 %)
Any suicidal thoughts or attempts	
Yes	22 (26 %)
No	64 (74 %)
Met symptom criteria for PTSD	
Yes	49 (57 %)
No	37 (43 %)
Met symptom criteria for anxiety	
Yes	28 (33 %)
No	58 (67 %)

## Discussion

This study offers unique insights into the characteristics of pregnancy terminations among women with SVRPs in eastern DRC. The results show that most terminations were performed using medications or herbs obtained outside the formal healthcare sector. At the time of the study, a high percentage of women who terminated SVRPs met symptom criteria for PTSD, depression, and anxiety, similar to other studies on survivors of sexual violence [1, 28].

Among the methods of SVRP termination, the most commonly reported medication was quinine and the most commonly reported herb was cimpokolo, known by the scientific name Phytolacca dodecandra L’Hérit [39, 40]. While there are reports on the use of cimpokolo as an abortifacient, we were not able to find evidence on its mechanism, safety, or efficacy. In contrast, there is robust evidence on the use of misoprostol, mifepristone, and vacuum aspiration for safe abortion care [17, 41–43], yet only one woman in this study reported using misoprostol and only one woman reported using an instrument or procedure. Though mifepristone is not registered in the DRC, misoprostol is registered and is reportedly available in some health centers [19]. Further research to investigate whether mifepristone and misoprostol can be purchased in local pharmacies in DRC would be useful. Other studies have demonstrated that when access to safe and legalized abortion is limited, alternative methods such as quinine and herbs are used [21, 44]; thus, it is likely that limited access to evidence-based methods of pregnancy termination in eastern DRC contributed to the reliance on herbs and quinine in this study.

Similarly, only 18 percent of women accessed medicines to terminate an SVRP from a physician, nurse, or midwife. This could indicate that women prefer to access informal and private networks to find care, or it could reflect a lack of available providers and resources. While this study did not evaluate if the health care providers were skilled in providing abortion care or if the health centers were equipped to provide abortion services, a recent survey among health clinics in eastern DRC found that none reported providing abortion services [19]. Further, 17 % of women in this study reported pregnancy termination in the second trimester when more advanced medical and surgical care may be required [17]. Data from other settings with restricted abortion show increased morbidity and mortality when unsafe abortions are performed in the second trimester [21, 45]. Taken together, our study data suggest that attention be re-directed at improving the understanding, knowledge and availability of evidence-based methods of pregnancy termination in this context.

The primary physical complaints reported in this study were bleeding, abdominal pain, and vaginal discharge following the pregnancy termination. These symptoms are expected side effects from the termination of a pregnancy; however, nearly half of all participants reported seeking care for these symptoms. This could suggest that the symptoms were more severe than expected or could reflect a higher proportion of incomplete abortions in the setting of non-evidence based methods. Given the legal restrictions and stigma around terminations in DRC, women may have taken herbs or medicines to induce vaginal bleeding and then allowing them to access post-abortion care services. This should be investigated in future studies. The number of women who reported a fever (18 %) and hospitalization (4 %) suggest opportunities to improve the safety of pregnancy terminations in this context. This study is unable to draw conclusions about the circumstances of the termination for women reporting a complication or requiring hospitalization. This should be explored in future studies, along with the prevalence of severe complications from pregnancy termination in this setting.

Given the reliance on non-evidence based termination methods, it is not surprising that a proportion of women reported more than one termination attempt. In addition, unpublished data from the parallel parenting survey show that 9 % of women currently raising a child from an SVRP reported on-going pregnancies after an attempt to terminate that SVRP. This proportion is similar to findings in another study from DRC of sexual violence survivors with SVRPs [14]. However, the true proportion of women with an on-going pregnancy after attempting to terminate may be higher, as our parenting group sample did not include women with SVRPs who gave birth to a live child but were no longer living with and/or caring for the child. Future research should characterize the methods used in these attempted terminations and further explore barriers to accessing evidence-based and safe methods of pregnancy termination.

The findings of this study may be used to inform programming and policy related to SVRPs in this region and in other conflict-affected settings. Immediate care for survivors of sexual violence should include access to emergency contraception; yet, the majority of women in this study reported an SVRP resulting from sexual captivity. In this context it is unlikely that women would have had timely access to medical care or to emergency contraception, which is most effective within five days of unprotected intercourse. This does not detract from the need for future research and programming to focus on education on the use of and the preventative distribution of emergency contraception given the high prevalence of sexual violence. However, it also emphasizes the importance of comprehensive care for survivors who become pregnant from sexual violence, including access to safe abortion services, comprehensive post-abortion care, pregnancy care and safe childbirth, post-pregnancy care, and options including adoption and future family planning. While reproductive health kits often include emergency contraception and misoprostol for post-abortion care [46], efforts should be made to include evidence-based medicines for pregnancy termination as well. In addition to ensuring availability of the medical equipment, supplies and medications required to provide comprehensive reproductive health services, it is also essential that health care providers be trained to provide non-judgmental care for women who wish to terminate or who have terminated a pregnancy. Although the current study did not examine attitudes of health care providers towards termination, this remains an important question for future research.

Our study’s quantitative data on mental health and emotional responses among women who terminated SVRPs emphasize the need for widely available psycho-social services for sexual violence survivors in eastern DRC [28]. It is important to note that our study data cannot draw conclusions on the relationship between pregnancy termination and mental health outcomes. Other studies of women undergoing abortion found that few women developed PTSD following pregnancy termination, and among those that did, the trauma cited as responsible for the PTSD was unrelated to the abortion [47]. Symptoms of mental health conditions in this study population may be related to the underlying trauma of the sexual violence, particularly given that the majority of women were held in captivity and may have experienced severe trauma secondary to repeated assaults. A high prevalence of mental health symptoms has also been demonstrated among women raising children from SVRPs in DRC [28] and other sexual violence survivors in DRC [1, 31], suggesting that the mental health symptoms in this study may be unrelated to the pregnancy termination. Alternatively, mental health symptoms could result from experiences of stigma surrounding sexual violence or SVRPs. Regardless of the contributing factors, the high rates of mental health symptoms observed in this and other studies among sexual violence survivors in DRC emphasize the need for comprehensive mental health services for all sexual violence survivors, including women with SVRPs.

### Limitations

Study results must be considered in the context of the study methodology and subject matter. Although the study achieved a relatively large sample size given the sensitive topic, its premature termination due to security concerns meant the study did not achieve the level of recruitment needed to apply RDS corrections, allowing it to be representative of the underlying population. Thus, the sample is closer to a snowball sample and the results cannot be generalized to the entire population. There may have been inherent selection biases that resulted in women who terminated using particular medications or herbs having been more likely to recruit women who used similar methods to terminate SVRPs.

The sensitive nature of this topic in a country where termination is highly restricted may have influenced participant responses and a social desirability bias may be present. Although care was taken to ensure participant privacy and security, it is possible respondents may have been less likely to indicate they used certain methods of termination, or to indicate who helped them terminate the SVRP. In addition, participants may be subject to recall bias given the retrospective nature of the study.

The majority of our study participants lived in an urban area, Bukavu, which may limit the generalizability of our findings. In addition, few participants reported civilian perpetrated sexual violence, though we do not have reason to suspect that the methods of pregnancy terminations would vary by perpetrator type.

Finally, mental health outcomes were assessed using symptom criteria rather than diagnostic interviews. Further, we cannot draw conclusions on what factors influenced the mental health outcomes reported in this study.

## Conclusion

In this study in eastern DRC, women who terminated an SVRP did so primarily with herbs and medications. Few women used evidence-based methods of pregnancy termination or sought assistance from within the formal healthcare sector. This suggests opportunities to explore and improve access to evidence-based methods of pregnancy termination for women with SVRPs in eastern DRC.

## Abbreviations

DRC: Democratic Republic of Congo
PTSD: Post Traumatic Stress Disorder
RDS: Respondent Driven Sampling
SVRP: Sexual violence related pregnancy

## References

[1] Johnson K, Scott J, Rughita B, Kisielewski M, Asher J, Ong R (2010). Association of sexual violence and human rights violations with physical and mental health in territories of the eastern Democratic Republic of Congo. JAMA.

[2] Kalonda JC (2012). Sexual violence in Congo-Kinshasa: necessity of decriminalizing abortion. Rev Med Brux.

[3] Linden JA (2011). Clinical practice. Care of the adult patient after sexual assault. N Engl J Med.

[4] Inter-Agency Working Group on Reproductive Health in Crises. Inter-agency Field Manual on Reproductive Health in Humanitarian Settings. 2010. http://www.who.int/reproductivehealth/publications/emergencies/field_manual_rh_humanitarian_settings.pdf. Accessed 24 Feb 2016. 26203479

[5] Cybulska B (2013). Immediate medical care after sexual assault. Best Pract Res Clin Obstet Gynaecol.

[6] Muhlstein J, Martrille L, Guillet-May F, Routiot T, Coudane H, Judlin P (2013). Post-rape pregnancy. Gynecologie, Obstetrique Fertilite.

[7] Steiner B, Benner MT, Sondorp E, Schmitz KP, Mesmer U, Rosenberger S (2009). Sexual violence in the protracted conflict of DRC programming for rape survivors in South Kivu. Confl Health.

[8] Bartels SA, Scott JA, Leaning J, Kelly JT, Joyce NR, Mukwege D (2012). Demographics and care-seeking behaviors of sexual violence survivors in South Kivu province, Democratic Republic of Congo. Disaster Med Public Health Prep.

[9] Tayler-Smith K, Zachariah R, Hinderaker SG, Manzi M, De Plecker E, Van Wolvelaer P (2012). Sexual violence in post-conflict Liberia: survivors and their care. Trop Med Int Health.

[10] The Reproductive Health in Conflict Consortium (2004). Emergency Contraception for Conflict-Affected Settings.

[11] Bartels S, Scott J, Leaning J, Kelly J, Joyce N, Mukwege D et al. Demographics and Care Seeking Behaviors of Sexual Violence Survivors in South Kivu Province, Democratic Republic of Congo. Accepted for publication in: Disaster Medicine and Public Health Preparedness. 2012; In press. 10.1001/dmp.2012.66 23241471

[12] Bartels S, Scott J, Leaning J, Mukwege D, Lipton R, VanRooyen M (2010). Surviving sexual violence in Eastern Democratic Republic of Congo. J Int Women’s Studies.

[13] Kelly JT, Betancourt TS, Mukwege D, Lipton R, Vanrooyen MJ (2011). Experiences of female survivors of sexual violence in eastern Democratic Republic of the Congo: a mixed-methods study. Confl Health.

[14] Dossa NI, Zunzunegui MV, Hatem M, Fraser W (2014). Fistula and other adverse reproductive health outcomes among women victims of conflict-related sexual violence: a population-based cross-sectional study. Birth.

[15] Holmes MM, Resnick HS, Kilpatrick DG, Best CL (1996). Rape-related pregnancy: estimates and descriptive characteristics from a national sample of women. Am J Obstet Gynecol.

[16] Black B, Bouanchaud P, Bignall J, Simpson E, Gupta M (2014). Reproductive health during conflict. Obstetrician Gynaecologist.

[17] World Health Organization. Clinical practice handbook for safe abortion. World Health Organization: 2014. 24624482

[18] Reproductive Health Access and Services in Emergencies (RAISE). Safe Abortion in Emergencies: Democratic Republic of Congo. New York: Columbia University Mailman School of Public Health; 2014.

[19] Casey SE, Chynoweth SK, Cornier N, Gallagher MC, Wheeler EE (2015). Progress and gaps in reproductive health services in three humanitarian settings: mixed-methods case studies. Confl Health.

[20] United Nations. World Abortion Policies 2011: United Nations, Department of Economic and Social Affairs, Population Division2011.

[21] Grimes DA, Benson J, Singh S, Romero M, Ganatra B, Okonofua FE (2006). Unsafe abortion: the preventable pandemic. Lancet.

[22] Dragoman M, Sheldon WR, Qureshi Z, Blum J, Winikoff B, Ganatra B (2014). Overview of abortion cases with severe maternal outcomes in the WHO Multicountry Survey on Maternal and Newborn Health: a descriptive analysis. BJOG.

[23] Singh S, Wulf D, Hussain R, Bankole A, Sedgh G (2009). Abortion Worldwide: A Decade of Uneven Progress.

[24] Heckathorn DD (1997). Respondent-driven sampling: a new approach to the study of hidden populations. Soc Probl.

[25] Heckathorn DD (2002). Respondent-driven sampling II: deriving valid population estimates from chain-referral samples of hidden populations. Soc Probl.

[26] Heckathorn DD (2007). Extensions of Respondent-Driven Sampling: Analyzing Continuous Variables and Controlling for Differential Recruitment. Sociol Methodol.

[27] Greiner AL, Albutt K, Rouhani SA, Scott J, Dombrowski K, VanRooyen MJ (2014). Respondent-Driven Sampling to Assess Outcomes of Sexual Violence: A Methodological Assessment. Am J Epidemiol.

[28] Scott J, Rouhani S, Greiner A, Albutt K, Kuwert P, Hacker MR (2015). Respondent-driven sampling to assess mental health outcomes, stigma and acceptance among women raising children born from sexual violence-related pregnancies in eastern Democratic Republic of Congo. BMJ Open.

[29] Rouhani SA, Scott J, Greiner A, Albutt K, Hacker MR, Kuwert P (2015). Stigma and Parenting Children Conceived From Sexual Violence. Pediatrics.

[30] KoBoToolbox. KoBoToolbox. Cambridge, MA. 2012. http://www.kobotoolbox.org.

[31] Bass JK, Annan J, McIvor Murray S, Kaysen D, Griffiths S, Cetinoglu T (2013). Controlled trial of psychotherapy for Congolese survivors of sexual violence. N Engl J Med.

[32] Pham PN, Weinstein HM, Longman T (2004). Trauma and PTSD symptoms in Rwanda: implications for attitudes toward justice and reconciliation. JAMA.

[33] Londono A, Romero P, Casas G (2012). The association between armed conflict, violence and mental health: a cross sectional study comparing two populations in Cundinamarca department, Colombia. Confl Health.

[34] Arroll B, Goodyear-Smith F, Crengle S, Gunn J, Kerse N, Fishman T (2010). Validation of PHQ-2 and PHQ-9 to screen for major depression in the primary care population. Ann Fam Med.

[35] Monahan PO, Shacham E, Reece M, Kroenke K, Ong’or WO, Omollo O (2009). Validity/reliability of PHQ-9 and PHQ-2 depression scales among adults living with HIV/AIDS in western Kenya. J Gen Intern Med.

[36] Kroenke K, Spitzer R, Williamas J (2007). Anxiety disorders in primary care: prevalence, impairment, comorbidity and detection. Ann Intern Med.

[37] Weathers FW, Litz BT, Herman DS, Huska JA, Keane TM. The PTSD Checklist (PCL): Reliability, validity, and diagnostic utility. 9th Annual Conference of the ISTSS; San Antonio, TX. 1993.

[38] Salganik MJ (2006). Variance estimation, design effects, and sample size calculations for respondent-driven sampling. J Urban Health.

[39] Royal Museum for Central Africa. Prelude Medicinal Plants Database: Phytolacca dodecandra L’Hérit. 2015. http://www.africamuseum.be/collections/external/prelude/view_plant?pi=10010. Accessed October 1, 2015.

[40] Chifundera K (1998). Livestock Diseases and the Traditional Medicine in the Bushi Area, Kivu Province. Democratic Repoblic of Congo.

[41] Lohr PA, Fjerstad M, DeSilva U, Lyus R. Abortion. BMJ. 2014;348. doi:10.1136/bmj.f7553.

[42] International Federation of Gynecology and Obstetrics, cartographer Consensus statement on uterine evacuation 2011.

[43] Dzuba IG, Winikoff B, Pena M (2013). Medical abortion: a path to safe, high-quality abortion care in Latin America and the Caribbean. Eur J Contracept Reprod Health Care.

[44] Adinma ED, Adinma JI, Iwuoha C, Akiode A, Oji E, Okoh M (2012). Knowledge and practices among medical abortion seekers in southeastern Nigeria. Southeast Asian J Trop Med Public Health.

[45] Harris LH, Grossman D (2011). Confronting the challenge of unsafe second-trimester abortion. Int J Gynaecol Obstet.

[46] Inter-agency Working Group on Reproductive Health in Crises (2011). Inter-Agency Reproductive Health Kits for Crisis Situations.

[47] Wallin Lundell I, Georgsson Ohman S, Frans O, Helstrom L, Hogberg U, Nyberg S (2013). Posttraumatic stress among women after induced abortion: a Swedish multi-centre cohort study. BMC Women’s Health.

